# Microarray-Based Analysis of Differential Gene Expression between Infective and Noninfective Larvae of *Strongyloides stercoralis*

**DOI:** 10.1371/journal.pntd.0001039

**Published:** 2011-05-03

**Authors:** Roshan Ramanathan, Sudhir Varma, José M. C. Ribeiro, Timothy G. Myers, Thomas J. Nolan, David Abraham, James B. Lok, Thomas B. Nutman

**Affiliations:** 1 Laboratory of Parasitic Diseases, National Institute of Allergy and Infectious Diseases, National Institutes of Health, Bethesda, Maryland, United States of America; 2 Bioinformatics and Computational Biosciences Branch, National Institutes of Health, Bethesda, Maryland, United States of America; 3 Laboratory of Malaria and Vector Research, National Institute of Allergy and Infectious Diseases National Institutes of Health, Bethesda, Maryland, United States of America; 4 Microarray Research Facility, National Institutes of Health, Bethesda, Maryland, United States of America; 5 Department of Pathobiology, School of Veterinary Medicine, University of Pennsylvania, Philadelphia, Pennsylvania, United States of America; 6 Department of Microbiology and Immunology, Thomas Jefferson University, Philadelphia, Pennsylvania, United States of America; University of Pittsburgh, United States of America

## Abstract

**Background:**

Differences between noninfective first-stage (L1) and infective third-stage (L3i) larvae of parasitic nematode *Strongyloides stercoralis* at the molecular level are relatively uncharacterized. DNA microarrays were developed and utilized for this purpose.

**Methods and Findings:**

Oligonucleotide hybridization probes for the array were designed to bind 3,571 putative mRNA transcripts predicted by analysis of 11,335 expressed sequence tags (ESTs) obtained as part of the Nematode EST project. RNA obtained from *S. stercoralis* L3i and L1 was co-hybridized to each array after labeling the individual samples with different fluorescent tags. Bioinformatic predictions of gene function were developed using a novel cDNA Annotation System software. We identified 935 differentially expressed genes (469 L3i-biased; 466 L1-biased) having two-fold expression differences or greater and microarray signals with a p value<0.01. Based on a functional analysis, L1 larvae have a larger number of genes putatively involved in transcription (p = 0.004), and L3i larvae have biased expression of putative heat shock proteins (such as *hsp-90*). Genes with products known to be immunoreactive in *S. stercoralis*-infected humans (such as *SsIR* and *NIE*) had L3i biased expression. Abundantly expressed L3i contigs of interest included *S. stercoralis* orthologs of cytochrome oxidase *ucr 2.1* and *hsp-90*, which may be potential chemotherapeutic targets. The *S. stercoralis* ortholog of fatty acid and retinol binding protein-1, successfully used in a vaccine against *Ancylostoma ceylanicum*, was identified among the 25 most highly expressed L3i genes. The sperm-containing glycoprotein domain, utilized in a vaccine against the nematode *Cooperia punctata*, was exclusively found in L3i biased genes and may be a valuable *S. stercoralis* target of interest.

**Conclusions:**

A new DNA microarray tool for the examination of *S. stercoralis* biology has been developed and provides new and valuable insights regarding differences between infective and noninfective *S. stercoralis* larvae. Potential therapeutic and vaccine targets were identified for further study.

## Author Summary

*Strongyloides stercoralis* is a soil-transmitted helminth that affects an estimated 30–100 million people worldwide. Chronically infected persons who are exposed to corticosteroids can develop disseminated disease, which carries a high mortality (87–100%) if untreated. Despite this, little is known about the fundamental biology of this parasite, including the features that enable infection. We developed the first DNA microarray for this parasite and used it to compare infective third-stage larvae (L3i) with non-infective first stage larvae (L1). Using this method, we identified 935 differentially expressed genes. Functional characterization of these genes revealed L3i biased expression of heat shock proteins and genes with products that have previously been shown to be immunoreactive in infected humans. Genes putatively involved in transcription were found to have L1 biased expression. Potential chemotherapeutic and vaccine targets such as *far-1*, *ucr 2.1* and *hsp-90* were identified for further study.

## Introduction

*Strongyloides stercoralis* is a parasitic nematode endemic to the tropics and subtropics that infects an estimated 30–100 million people worldwide. Chronically infected individuals have the potential to develop hyperinfection syndrome or disseminated disease, clinical entities that carry a very high (87–100%) mortality if unrecognized [1].

Free-living *S. stercoralis* infective third stage (L3i) larvae residing in the soil penetrate intact skin and blood vessels, ultimately developing to adults in the small intestine. Adult females, typically residing in the duodenum of the host, produce eggs by mitotic parthenogenesis that develop into first-stage (L1) larvae that are excreted into the stool. L1 larval progeny of parasitic females develop into free-living adults unless triggered by genetic, environmental, or host factors to develop directly into L3i larvae [2], [3]. Despite sharing many characteristics, L1 and L3i larvae can be distinguished by their behavior and morphology. L1 larvae have a short, trilobed pharynx and expend much of their energy on feeding and growth [3]. L3i larvae, by contrast, can survive in harsh environmental conditions, enabled by a comparatively thickened cuticle, constricted gastrointestinal tract, and closed mouth. These larvae are developmentally arrested, non-feeding, stress resistant, and long lived [3]–[5].

A high degree of specificity between these stages has been suggested by expressed sequence tag (EST) based analysis of free living L1 and L3i larvae for *S. stercoralis* [6]–[8]. These comparisons, however, are based on short reads of cDNA libraries and assumptions about abundance. There remain many unanswered questions about the basic molecular features underlying the apparent morphologic and behavioral differences between these larval stages. An improved understanding of these differences can provide insights into what defines infectivity and may ultimately prove useful in defining targets for the development of vaccines and therapeutics against this parasite.

In order to answer these questions, a DNA microarray tool for *S. stercoralis* – the species causing the vast majority of human infection worldwide - is needed. Although a DNA microarray has recently been developed for *Strongyloides ratti*, the natural parasite of brown rats (Rattus norvegicus) [9], previous work has suggested little conservation of gene expression profiles between these two species [10], underscoring the need for a DNA microarray specific to this species.

The availability of a *S. stercoralis* DNA microarray enables comparative analyses across nematodes, which can be utilized to further our understanding of the biologic determinants of parasitism. The free-living, non-parasitic, nematode *C. elegans* has been used as a model species for comparison with *S. stercoralis*. *C. elegans* dauer stage larvae and *S. stercoralis* L3i larvae share many morphologic and physiologic characteristics. The ‘dauer hypothesis’ recognizes these similarities and suggests that the same molecular genetic mechanisms control the morphogenesis of these stages [11]. Comparative genomics of gene expression based on EST abundance data for *S. stercoralis* suggests a higher degree of similarity between *S. stercoralis* L1 and *C. elegans* non-dauer expressed genes [6]. By contrast, a robust ‘dauer-L3i expression signature’ has not been found [6]. A comparative analysis based on microarray expression data for these species could prove useful not only in identifying a ‘dauer-L3i expression signature’ should it exist, but also in uncovering potentially significant determinants of *S. stercoralis* L3i infectivity.

The purpose of this study was to: 1) develop and optimize a DNA microarray tool for *S. stercoralis*, 2) utilize this microarray to examine differences in gene expression between L3i and L1 larvae and 3) perform a comparative microarray analysis between parasitic *S. stercoralis* and non-parasitic *C. elegans* in order to develop further insights into the biologic determinants of parasitism.

## Methods

### Ethics statement

Animal handling and experimental procedures were undertaken in compliance with the University of Pennsylvania's Institutional Animal Care and Use Committee (IACUC) guidelines. Ethical approval was obtained for the study (protocol number 702342) from IACUC (University of Pennsylvania, Philadelphia, PA).

### Parasites

All larvae used in this analysis were obtained from laboratory dogs infected with *S. stercoralis*, UPD strain [12]. Fecal samples from dogs were processed using the charcoal coproculture followed by Baermann funnel technique, as outlined elsewhere [13]. Post parasitic L1 larvae were recovered from freshly deposited stool samples; L3i larvae were recovered after 7 days of stool incubation at 25°C. L3i larvae underwent surface decontamination by migration through low-melting-point agarose. L1 larvae were decontaminated by 3 washes with phosphate buffered saline (PBS) containing an antibiotic cocktail. Decontaminated parasites were subsequently stored in Trizol reagent (Invitrogen, San Diego, CA) at −80°C. Using this method, 30,700 post-parasitic L1 and 50,000 L3i *larvae* were collected.

### Isolation of total RNA from larvae

Total RNA was extracted by thawing pooled samples of L1 and L3i larvae at 37°C in a warm water bath and centrifuging the samples at 4°C (805× g) for 10 minutes to obtain a pellet. The pellet was frozen in liquid nitrogen, ground thoroughly with an autoclaved mortar and pestle and then purified using an RNeasy mini kit (Qiagen, Valencia, CA) following the manufacturer's protocol. A Nano Drop-1000 spectrophotometer (NanoDrop Products, Wilmington DE) was used to determine the RNA concentration in each sample. RNA was more precisely quantified and quality assessed using the 2100 Bioanalyzer (Agilent, Santa Clara, CA).

### Amplification and labeling

RNA samples from L1 and L3i stage larvae were co-hybridized using Cy3 and Cy5 labels to discriminate the relative level of target bound to the microarray probe. Fluorescent-labeled cDNA targets were prepared from total RNA using the Ovations amino-allyl kit (NuGEN, San Carlos, CA) according to the manufacturer's protocol. The kit utilizes an oligo dT primer for selective amplification of mRNA transcripts.

### Hybridization procedure

Labeled samples were combined with blocking components poly(dA), yeast tRNA, and human Cot-1, in hybridization buffer composed of 25% formamide/5× saline-sodium citrate (SSC)/0.2% (w/v) sodium dodecyl sulfate (SDS) to a total volume of 60 µl. After heating the sample (95°C for 3 minutes), it was centrifuged (20,000× g) for 3 minutes. Fifty eight µl of the sample (1.6 µg of labeled cDNA) was loaded onto the microarray chip. The microarray chips were hybridized overnight at 45°C using the MicroArray User Interface (MAUI) hybridization system (BioMicro Systems, Inc., Salt Lake City, UT). The following day, the chips were washed twice in 1× SSC/0.05% (w/v) SDS buffer (3 minutes each wash) and twice in 0.1× SSC buffer (5 minutes each wash).

For the present study, four technical replicate experiments using pooled L1 and L3i larvae were performed, including one dye swap. The microarray chips were imaged using a GenePix 4000 B scanner (Molecular Devices, Sunnyvale, CA). Agilent Feature Extraction software was used for image analysis, protocol GE2-v5 10 Apr08. The data discussed in this publication have been deposited in the National Center for Biotechnical Information (NCBI) Gene Expression Omnibus (GEO) and are accessible through GEO Series accession number GSE24735 (http://www.ncbi.nlm.nih.gov/geo/query/acc.cgi?acc=GSE24735).

### Microarray design

ESTs (11,335) were identified from L1 and L3i cDNA libraries created as part of the nematode EST project [6], [7]. ESTs were organized into 3,571 contigs by bioinformatics analysis [14]. Oligonucleotide probes designed to hybridize with these contigs were used to develop early versions (V1 and V2) of chips manufactured by Combimatrix (Irvine, CA) based on a variety of algorithms for oligonucleotide design. Versions 1 and 2 were assessed for performance using RNA from L1 and L3i larvae. After testing the performance of these two versions of the arrays, an optimized version (V3) was developed. The best probe for each target was selected based on the average signal intensity for all arrays and the number of arrays with detectable signal. The spot density was 22K spots per array. Of the six oligonucleotides designed per target, one was designed using the Array Designer program (Premier Biosoft International, Palo Alto, CA), two were designed using E-Array (Agilent, Santa Clara, CA) using the “base composition” method (replicated twice), two were designed using E-array “best Tm” method, and the last was a 40-mer designed using Array Designer. Probes were selected to avoid cross-hybridization to other sequences in the target (contig) dataset manufactured by Agilent SurePrint. The probes designed to make the V3 microarray are found in Table S1 in Supporting Information Text S1.

### Functional annotation

All data were exported into the cDNA Annotation System (dCAS) [14], [15]. This tool enabled annotation of each *S. stercoralis* contig based on Basic Local Alignment Search Tool (BLAST) alignments against multiple databases (NCBI nr protein database (NR), Gene Ontology (GO), euKaryotic Orthologous Groups (KOG), Pfam protein families database (PFAM), Simple Modular Architecture Research Tool (SMART), Wormbase (CELEG), and Saccharomyces genome database (YEAST) and provided the corresponding E-values. The database was also annotated manually with a composite categorization that summarized the findings across databases. The entire annotated database, with hyperlinks to the NIAID exon website, is accessible for download at: http://exon.niaid.nih.gov/transcriptome/S_stercoralis/SS-Supp-Web.zip. A stand-alone version can also be accessed and downloaded at: http://exon.niaid.nih.gov/transcriptome/S_stercoralis/SS-Supp-StandAlone.zip. Extract the excel file and the links directory to your own computer for browsing the hyperlinks locally.

### Statistical analysis

Spot values were calculated using a linear lowess dye normalization. Further, the 50^th^ percentile of a set containing all the ribosomal genes in the array was applied to all spot values. In cases of multiple spots for the same *S. stercoralis* contig, the average of the log_2_ signal was calculated for each array. The mean signal ratio (log_2_ L3i/L1) was calculated from the signals for all 4 arrays. No surrogate values were applied. A single group *t*-test analysis was calculated on the data set. Variance shrinkage was not used when calculating p-values for differential expression. Differentially expressed genes were identified using a ‘cutoff’ of 2 fold expression difference or greater for log_2_ L3i/L1 signal ratios, and p<0.01 for microarray signal data (false discovery rate (FDR) = 2.5%).

### Functional analysis

A functional analysis was performed based on annotations provided by each database (Pfam, SMART, KOG, etc.). The number of genes per functional category (e.g. transcription, cytoskeleton, metabolism, etc.) was compared between L1 and L3i differentially expressed genes (as defined by the above cutoff). To ascertain whether genes belonging to certain functional classes were more likely to be highly expressed in one stage or another, we used a statistical test for one proportion using Normal approximation. Assuming a null proportion of 0.5 (i.e., that there is no difference in the number of genes of that category for the two classes), p values were calculated for deviation from 0.5 using Normal approximation. P values were adjusted for multiple comparisons using the Bonferroni criterion.

### Gene-set enrichment analysis

Gene Set Enrichment Analysis (GSEA) is a robust method for analyzing molecular profiling data examines the clustering of a pre-defined group of genes (gene set) across the entire microarray database (all 3,571 contigs) in order to determine whether the gene set has biased expression in one larval stage versus another [16]. GSEA was used in this study to complement our use of single gene methods and determine whether *S. stercoralis* gene sets grouped according to various putative categories (for example, putative extracellular matrix genes) showed biased expression in either larval stage. For this analysis, the entire list of contigs on the microarray was sorted by mean log_2_ L3i/L1 signal ratios. The distribution of genes from an a priori defined gene set throughout this ranked list was then determined using GSEA. Based on this distribution, the expression difference for each gene in the set is aggregated and a p-value for significance of the gene set as a whole is calculated using the Kolmogorov-Smirnoff test.

**Figure 1:**
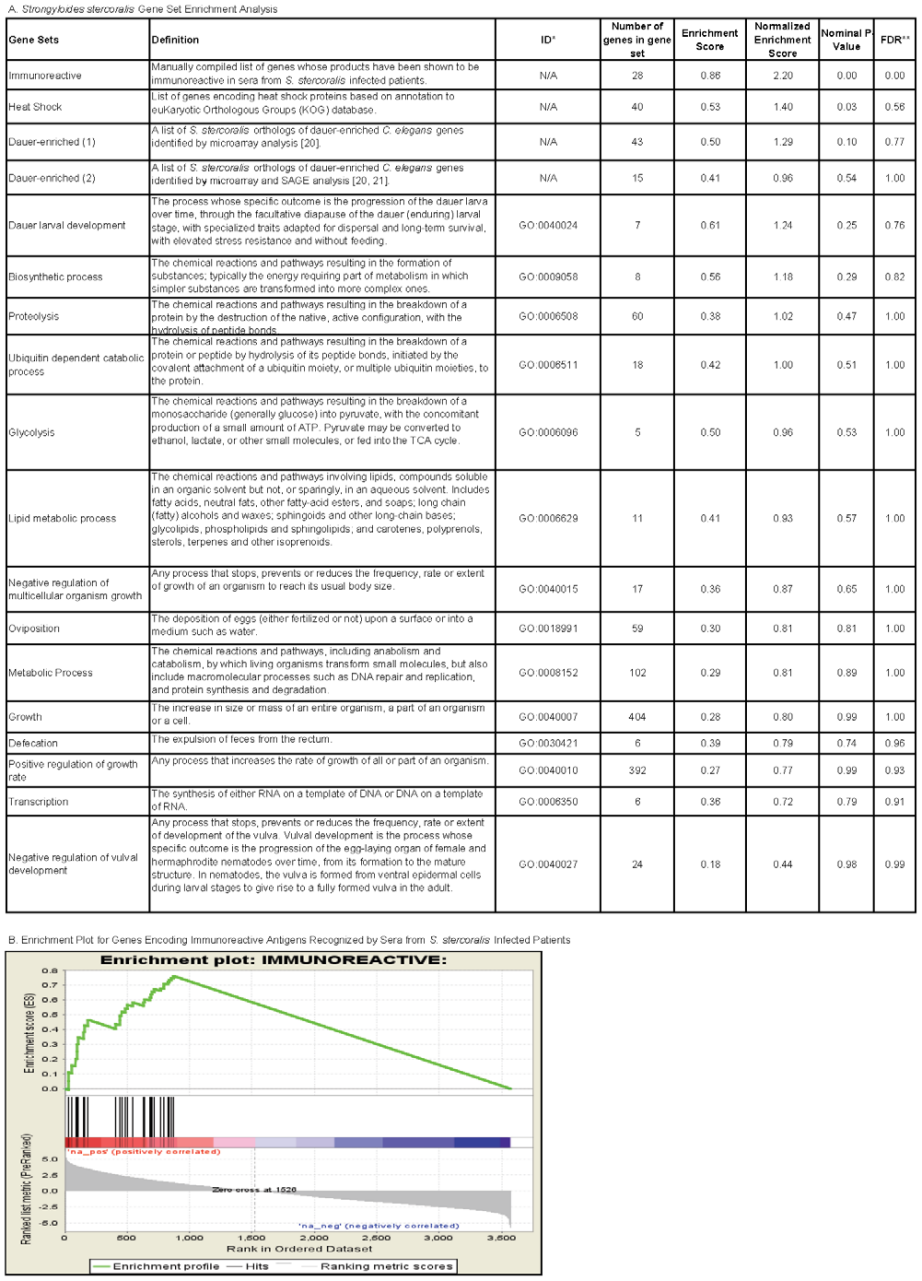
Gene Set Enrichment Analysis and enrichment plot. A. Gene sets were compiled by listing the *S. stercoralis* orthologs of *C. elegans* genes assigned to Gene Ontology (GO) categories (downloaded from www.wormbase.org). Some gene sets were manually compiled. Only gene sets with at least 5 *S. stercoralis* contigs were included in this analysis. The results of the GSEA are listed for each gene set. The enrichment score reflects the degree to which each gene set is represented at the top or bottom of the list of 3,571 contigs ranked by fold change (L3i enriched = more positive, L1 enriched = more negative). The normalized enrichment score accounts for differences in gene set size and can be used to compare results across gene sets. The nominal p value estimates the statistical significance of the enrichment for a single gene set and does not correct for gene set size and multiple hypothesis testing. *ID = Gene Ontology Identification. **The False Discovery Rate (FDR) is adjusted for gene set size and multiple hypotheses testing. B. This plot depicts the distribution of individual genes (vertical black lines) encoding immunoreactive antigens recognized by sera from patients infected with *S. stercoralis*. This gene sets was analyzed against a list of 3,571 *S. stercoralis* contigs ranked by fold change of log_2_ L3i/L1 mean signal ratios. The clustering of individual genes towards the left side of the list (above the red bar) suggests L3i-biased enrichment of this gene set. These genes are individually listed in Table S8 in Supporting Information Text S1.

Gene sets were compiled by first downloading GO categories from Wormbase (www.wormbase.org) for *C. elegans* genes. Definitions for each GO category used can be found at http://www.wormbase.org/db/ontology/gene. *S. stercoralis* orthologs for *C. elegans* genes were determined by dCAS based on BLAST alignments to the *C. elegans* gene. BLAST matches with E values>0.05 were excluded. Gene sets with fewer than 5 *S. stercoralis* contig matches were excluded from GSEA analysis. Using these criteria, 18 *S. stercoralis* gene sets were created (see Figure 1A). Additional manually compiled gene sets included the group of *S. stercoralis* genes whose products have been shown to be immunoreactive in humans infected with S. *stercoralis* [17]–[19], and a group of putatively identified heat shock proteins.

### Comparative microarray analysis of *S. stercoralis* and *C. elegans*

Microarray expression data for *S. stercoralis* L3i and *C. elegans* dauer larvae were compared using several methods as follows: 1) We defined three gene sets comprising the *S. stercoralis* orthologs of “dauer-enriched” *C. elegans* genes derived from either *C. elegans* microarray expression data alone, both serial analysis of gene expression (SAGE) and microarray expression data or from the Gene Ontology category dauer larval development (Figure 1A) [20], [21]. We then used GSEA to determine whether these gene sets showed significant L3i enrichment. 2) We examined whether a correlation exists between *C. elegans* dauer/L1 microarray expression data obtained by Wang and colleagues [20] with our *S. stercoralis* L3i/L1 microarray expression data. The previously obtained *C. elegans* microarray expression data can be found at http://cmgm.stanford.edu/~kimlab/dauer/ExtraData.htm, Table S1 in Supporting Information Text S1, column “AdjD/L1_Ratio” which corresponds to the average log_2_ expression values for *C. elegans* dauer larvae at time 0 relative to L1 larvae [20]. 3) Using these data, we calculated the absolute value of the difference between fold change values for *C. elegans* genes and their *S. stercoralis* orthologs (*C. elegans* dauer/L1 fold change - *S. stercoralis* L3i/L1 fold change). Only those genes with robust microarray expression data (p values<0.01) were included. In order to identify those genes that are expressed differently by *S. stercoralis* L3i and *C. elegans* dauer larvae, a list was generated of all *S. stercoralis-C. elegans* orthologs with the greatest differences in fold change values (absolute value >2). The list was further narrowed to include only those *S. stercoralis-C. elegans* gene pairs where gene expression was regulated in opposite directions between the two nematodes (Table 1).

**Table 1:** Differences between *S. stercoralis* (L3i/L1) and *C. elegans* (dauer/L1) gene expression profiles^a^.

			Fold change direction^b^		Fold change		
*C. elegans* match	*S.stercoralis* contig	Putative identification	*C. elegans*(dauer/L1)	*S. stercoralis*(L3i/L1)	*C. elegans*	*S. stercoralis*	Absolute difference^c^
C13D9.8	2626	*ncx-9*	↑	↓	3.59	0.39	3.20
T14D7.2	2474	protein_id:CAB03365	↑	↓	21.75	0.40	21.36
C06B3.4	2873	*stdh-1* estradiol 17 beta-dehydrogenase	↑	↓	3.71	0.44	3.27
T02D1.5	1790	pmp-4 ABC transporters	↑	↓	16.57	0.45	16.12
T19B10.2	504	protein_id:CAA98547	↑	↓	2.56	0.55	2.01
T04B2.5	2003	protein_id:CAA92628	↑	↓	3.97	0.56	3.41
F46C3.1	1269	*pek-1* eukaryotic translation initiation factor 2 alpha kinase PEK	↑	↓	3.61	0.57	3.04
F19H8.1	1652	*tps-2* trehalose phosphate synthase	↑	↓	4.22	0.65	3.57
F10B5.3	1998	Zinc finger, C2H2 type	↑	↓	4.60	0.66	3.94
Y57G11C.15	850	protein transport protein SEC61 alpha subunit	↑	↓	2.92	0.76	2.16
F42D1.2	118	tyrosine aminotransferase	↓	↑	0.17	2.22	2.05
F42D1.2	117	tyrosine aminotransferase	↓	↑	0.17	2.87	2.69
C53B4.5	2200	*col-119* collagen	↓	↑	0.10	2.98	2.88
F28C1.2	836	*egl-10* G-protein beta subunit GPB-2	↓	↑	0.78	2.99	2.21
B0491.5	291	protein_id:CAA90087	↓	↑	0.84	3.18	2.34
E02H1.7	3417	*nhr-19* Zinc finger, C4 type (two domains)	↓	↑	0.35	3.25	2.90
C02F12.7	64	*tag-278*	↓	↑	0.27	3.89	3.62
C53B4.5	2267	*col-119* collagen	↓	↑	0.10	4.11	4.01
E02H1.7	1846	*nhr-19* Zinc finger, C4 type (two domains)	↓	↑	0.35	4.18	3.83
C03B1.12	785	*lmp-1*	↓	↑	0.70	5.09	4.39
ZK863.2	9	*col-37* collagen status	↓	↑	0.11	6.74	6.63
B0365.3	220	*eat-6* Na(+)/K(+) ATPase alpha subunit	↓	↑	0.71	6.83	6.12
B0495.2	358	CDC2 status	↓	↑	0.71	8.28	7.58
F22B3.4	427	*nmy-2* myosin heavy chain status	↓	↑	0.89	8.33	7.44
F42D1.2	116	tyrosine aminotransferase	↓	↑	0.17	9.04	8.87

a Shown only are *S. stercoralis*-*C. elegans* orthologs with an absolute difference >2. All *S. stercoralis-C. elegans* orthologs are BLAST matches with an E value<0.05. The p value for *S. stercoralis* microarray signal data was <0.01.

b Arrows indicate whether genes had increased (↑) or decreased (↓) expression in *C. elegans* dauer or *S. stercoralis* L3i larvae relative to its respective L1 stage.

c The values in this column were calculated by taking the absolute value of [fold change *C. elegans* dauer/L1 - fold change *S. stercoralis* L3i/L1]. *C. elegans* expression data were previously obtained by Wang and colleagues [20].

### Microarray validation by quantitative real-time polymerase chain reaction (qPCR)

**Figure 2:**
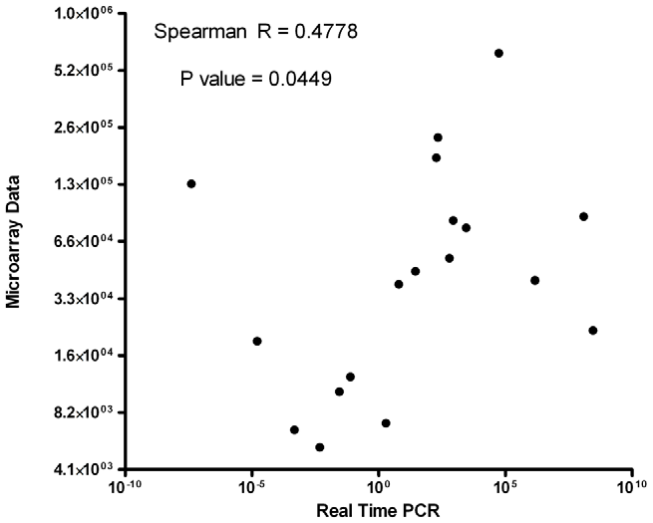
Correlation between microarray signal data and quantitative PCR data. Quantitative PCR was performed using primer probe sets designed from abundantly expressed L1 and L3i S. stercoralis contig sequences (L1 biased contigs 55, 222, 387, 2328; L3i biased contigs_24, 25, 65, 243, 2136) with cDNA synthesized from L1 and L3i larvae (n = 18). Each data point is the calculated negative delta C_T_ (sample C_T_ minus control C_T_) for the mean of 3 replicates. These data are plotted against the corresponding L1 or L3i average intensity microarray signal. A positive correlation was found (Spearman rank = 0.4778; p = 0.0449).

The sequences of L3i biased genes (contigs 24, 25, 65, 243, 2136) and L1 biased genes (contigs 55, 222, 387, 2328) were used to create primer-probe sets designed and manufactured by Applied Biosystems (Foster City, CA). The sequences for these primer probes are listed in Table S2 in Supporting Information Text S1. The *S. stercoralis* control genes for qPCR analysis was *S. stercoralis* glyceraldehyde 3 phosphate dehydrogenase (GAPDH; GenBank accession number BI773092; contig_90; log_2_L3i/L1 = −0.28179). Post-parasitic L1 and L3i larvae (distinct from those hybridized onto the microarray) were collected and total RNA made as described above. Total RNA (1 µg) from L1 and L3i larvae was used to synthesize cDNA. qPCR was performed using all 9 primer probe sets in separate reactions with L1 cDNA and also with L3i cDNA. The reaction was performed using 10× RT buffer (10 µl), 25 mM MgCl2 (22 µl), dNTP (20 µl), random hexamers (5 µl), RNase inhibitor (2 µl), and multiscribe reverse transcriptase (50 U/µl; 6.25 µl) in a microamp 96-well reaction plate (Applied Biosystems). De-ionized, distilled water was added to total volume of 65.25 µl. Cycling conditions were: 25°C for 10 minutes, 37°C for 60 minutes, 95°C for 5 minutes, then 4.0°C. Each experiment was performed in triplicate. The mean negative delta threshold cycle (delta C_T_) was calculated for each sample. The data generated by performing qPCR using primer probes for 9 contigs on L1 and L3i cDNA (n = 18) was plotted against the average L1 and L3i intensity signals for each gene (Figure 2).

## Results

### Identification of differentially expressed genes

**Figure 3:**
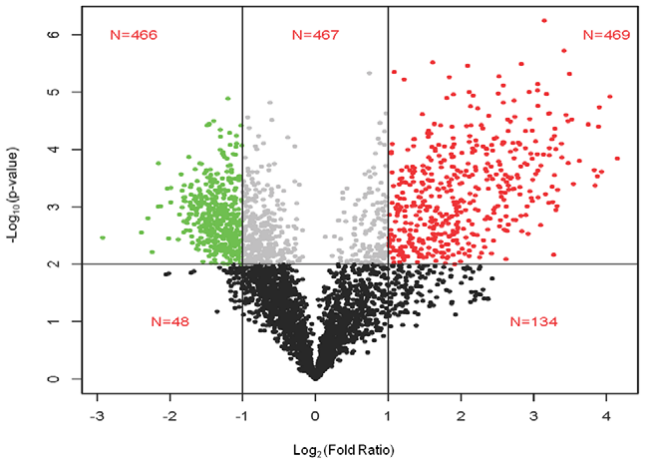
Volcano plot used in differential expression analysis. The *x*-axis is log_2_ ratio of gene expression levels between two stages; the *y*-axis is adjusted p value based on −log_10_. The colored dots (L1 = green) and right (L3i = red) represent the differentially expressed genes based on p<0.01 (False Discovery Rate = 2.5%; represented by black horizontal line) and 2-fold expression difference (represented by two black vertical lines).

A total of 3,571 distinct contigs were studied by this microarray analysis (Table S3 in Supplemental Information Text S1). Using pre-defined cutoffs, 935 contigs were identified as differentially expressed as shown in the volcano plot (Figure 3). Of these, 466 genes were L1 biased (Table S4 in Supporting Information Text S1) and 469 genes were L3i biased (Table S5 in Supporting Information Text S1). Among the 25 most highly expressed L3i genes were the *S. stercoralis* orthologs of fatty acid/retinol binding protein-1 (contig 1151; 11 fold expression difference), a ferritin chain homolog (contig 94; 14 fold expression difference), and one of four putative trehalases (contig 68; 14-fold expression difference). Among the 25 most highly expressed L1 genes were electron transport chain proteins such as NADH dehydrogenase (contig 371; 0.13-fold change); cytochrome b (contig 2328; 0.19 fold change) and cytochrome c oxidase subunit 1 (contig 55; 0.29 fold change). The 25 most highly expressed L1 or L3i genes are listed in Table S6 in Supporting Information Text S1.

### Functional analysis of L1 and L3i biased genes

**Figure 4:**
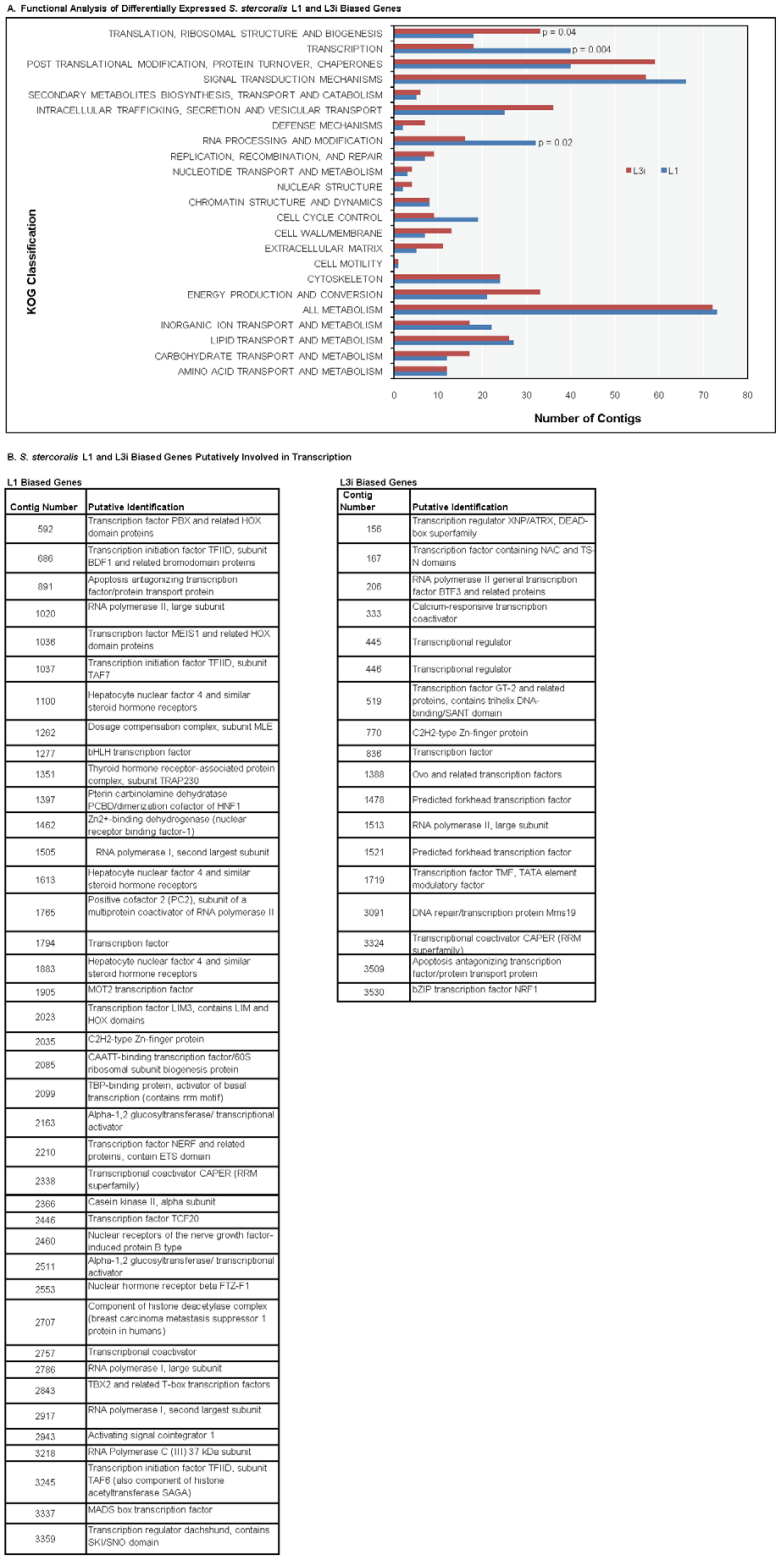
Functional analysis of differentially expressed *S. stercoralis* L1 and L3i genes. Horizontal bar graph (A) depicting the number of contigs per functional category in each stage (x axis), according to BLAST matches to the KOG database (y-axis). BLAST matches with E values>0.05 were excluded. The strongest difference was found between L1 and L3i genes putatively involved in transcription (p = 0.004). L1 = blue; L3i = red. The table (B) lists the L1 and L3i biased *Strongyloides* contigs putatively classified under “transcription.”

A greater number of L1 (n = 40) than L3i biased (n = 18) genes were putatively involved in transcription (p = 0.004, not Bonferroni adjusted; see Figure 4A,B). A complete listing of these genes is shown in Figure 4B. This finding was also noted in an analysis of classifications based on GO categories (p = 0.01 for ‘transcription’), and manual annotations (p = 0.007 for ‘transcription machinery’), although p values were not <0.05 when Bonferroni-adjusted for multiple comparisons. BLAST matches to SMART and Pfam databases both indicated that the sperm-containing glycoprotein (SCP) domain was found exclusively in the L3i-group (n = 13 genes; see Table S7 in Supporting Information Text S1 for the complete list; p value based on matches to Pfam = 0.003, Bonferroni-adjusted for multiple comparisons).

Of the entire 3,571 contigs, 1,351 *S. stercoralis* genes (37.8%) were of unknown function (manual annotation).

### GSEA

*S. stercoralis* orthologs were matched to 35 sets of *C. elegans* genes grouped by various categories (e.g. negative regulation of vulval induction, oviposition, heat shock proteins, etc.). Eighteen of 35 gene sets queried met criteria for inclusion into the GSEA analysis (based on minimum size of 5 genes; see Figure 1A). Of these 18 gene sets, only 2 gene sets were significantly enriched in the L3i phenotype at nominal p value<5%. The most significantly enriched genes were those with immunoreactive gene products recognized by sera from infected individuals (Figure 1B; nominal p-value<0.0001; FDR<0.0001). Heat shock proteins were the next most highly enriched (nominal p value = 0.034, FDR = 0.56). For an annotated list of the individual genes enriched in each of these categories, refer to Tables S8 and S9 in Supporting Information Text S1. None of the 18 gene sets were enriched in the L1 phenotype.

### Comparative microarray analysis of *S. stercoralis* and *C. elegans*

Four hundred and twenty two of 3,571 *S. stercoralis* contigs had *C. elegans* orthologs for which robust microarray signal data were available. When *C. elegans* and *S. stercoralis* microarray signals were plotted against each other, a poor and non-significant correlation was found (Spearman rank = 0.06; p = 0.2444, graph not shown). No significant L3i enrichment of *S. stercoralis* orthologs of *C. elegans* ‘dauer enriched’ genes was found by GSEA (nominal p-value = 0.10). On the contrary, 25 orthologs expressed in opposite directions by dauer and L3i larvae relative to their respective L1 stage larvae were identified (see Table 1).

### Correlation between EST and microarray data

A statistically significant positive correlation was found between microarray expression data and EST abundance data (p<0.0001; max R^2^ = 0.26; graph not shown).

### Validation of microarray data with qPCR

A positive correlation was found (Spearman rank = 0.4778; p = 0.0449) between average L1 or L3i microarray intensity signals and mean negative delta C_T_ of qPCR (Figure 2).

## Discussion

In this microarray based analysis of differential gene expression between infective and noninfective *S. stercoralis* larvae, we uncovered differences in the expression of genes putatively encoding transcription factors, heat shock proteins and antigens known to be immunoreactive in sera from infected humans. A comparative microarray analysis of our data revealed several differences between *S. stercoralis* L3i and *C. elegans* dauer stage larvae, such as in the expression of genes putatively encoding collagen and myosin. Potential therapeutic and vaccine targets were identified for further study.

### L1 larvae appear to be transcriptionally more active

Analogous to their non-dauer *C. elegans* counterparts, actively growing *S. stercoralis* L1 larvae are thought to have higher rates of transcription relative to L3i-stage larvae. This supposition is based on comparisons between *C. elegans* non-dauer biased genes and *S. stercoralis* L1-biased genes that suggest transcriptional conservation of genes involved in early larval growth [6]. Consistent with this finding, we found L1 biased expression of genes putatively involved in transcription. Among the *S. stercoralis* L1-biased genes involved in transcription were transcription initiation factors (contigs 3245, 1037, 686), transcription factors (contigs 1905, 1277, 891, 2023, 2446, 1036, 1794, 592, 2210), and subunits of RNA polymerase (contigs 1505, 3218, 1020, 2917). By contrast, the L3i-biased genes involved in transcription though fewer, included transcriptional regulators (contigs 446, 445, 156) as well as transcription factors (contigs 1521, 519, 836, 167, 1478), implying that L3i larvae are not transcriptionally inactive and may regulate transcription differently. This would be consistent with what is known of *C. elegans* dauer larvae, which express distinct sets of dauer-specific genes at certain time points (dauer exit, for example) [20], [21].

### L3i biased expression of genes with products that have been shown to be immunoreactive in *S. stercoralis*-infected humans

Not surprisingly, genes encoding *S. stercoralis* antigens known to produce robust antibody responses in infected humans were found to have L3i biased expression by GSEA [17]–[19]. Two of these genes, IgG immunoreactive antigen (SsIR) and NIE antigen, have been recently employed in serodiagnostic assays with some advantage over crude antigen [19]. The finding that genes with products capable of inducing protective immunity demonstrate stage-biased gene expression supports the further investigation of these genes as vaccine candidates.

Heat shock proteins have been shown to play a critical role in determining parasite survival during stressful conditions because they can bind denatured or misfolded proteins [22], [23]. Biased expression of genes encoding heat shock proteins in the *S. stercoralis* L3i relative to L1 larvae, as suggested by GSEA, is consistent with this role. *Hsp-90* in particular has been identified as a parasitism-central gene based on changes in *S. ratti* gene expression during high immune pressure [22] and is similarly abundantly expressed by *S. stercoralis* L3i larvae.

### Sperm containing glycoprotein (SCP) domain exclusively found in L3i larvae

The SCP domain, found exclusively in L3i biased genes, is a conserved domain of unknown function present in a wide range of organisms [24]. Interestingly, it has been found to be present in activation-associated secreted proteins that have been studied as potential vaccine targets in other nematodes [24], [25]. Whether overrepresentation of the SCP domain in the L3i group is related to the presence of these secreted proteins is unclear, but activation-associated secreted proteins have been found to be important in many parasitic nematodes in which they have been studied to date.

### *C. elegans* dauer and *S. stercoralis* L3i larvae have distinct characteristics

Consistent with previous findings, a striking L3i-*C. elegans* ‘dauer expression signature’ was not uncovered in this comparative microarray analysis [6]. We instead identified genes that are regulated in apparently opposite manners by *C. elegans* dauer and *S. stercoralis* L3i larvae which offer useful clues about the biology of *S. stercoralis* parasitism. L3i biased expression of the putative *nmy-2* gene (encoding the myosin heavy chain) is consistent with the highly motile nature of L3i larvae which, unlike their dauer counterparts, seek out and initiate infection in a host. Although dauer and L3i larvae both contain a cuticle that enables survival in the environment, the parasitic cuticle has been associated with the ability of infective stages to evade the immune response of the host, and its structure varies from one species to another [26]. Biased expression of genes putatively encoding particular collagens (*col-37*, *col-119*) in the L3i but not the *C. elegans* dauer, points to differences in the composition of the parasitic cuticle that could potentially have a role in this regard. In fact, a recent microarray based analysis of the response of the *S. ratti* transcriptome to host immunologic environment notes upregulation of collagen genes by *S. ratti* which is believed to play a protective role for the parasite [27].

*C. elegans* dauer and *S. stercoralis* L3i larvae can survive in the environment even in the absence of a steady source of food. One way by which this occurs is by the development of electron-dense intestinal granules that store non-lipid products [11]. The gene *lmp-1* plays an essential role in this regard for dauer larvae as suggested by RNA interference studies [28]. It is likely that L3i larvae similarly utilize these granules while in the environment. The presence of these granules may additionally explain the darkened color of the radially constricted intestines of L3i larvae, an appearance shared by its dauer counterpart.

A key feature shared by dauer and L3i larvae is the ability to extend the lifespan while in the free-living state. In both *C. elegans* and *S. stercorali*s, the forkhead transcription factor DAF-16 plays a role in regulating dauer diapause, longevity and metabolism [11], [29], [30]. A downstream target of DAF-16, *egl-10*, is known to be negatively regulated by DAF-16 in *C. elegans* [29]. By contrast, this gene was found to have biased L3i larval expression in *S. stercoralis*. Such discordance is consistent with findings from a prior study that failed to detect a transcriptional profile typical of down-regulated insulin-like signaling in long-lived parasitic females of *S. ratti* [31]. Although the downstream targets of insulin-like signaling have not been fully elucidated in *Strongyloide*s species, the apparent upregulation of *Ss-egl-10* in the L3i potentially highlights adaptations at a molecular level that likely underlie the evolution to parasitism. Such adaptations could include alterations in genes controlling metabolic and developmental functions, adaptations of pre-existing genes to encode new functions, and gene duplication and diversification [32]. The apparent lack of a *C. elegans* dauer-like transcriptional profile in *S. stercoralis* L3i is also consistent with published findings on the expression of transcripts encoding the orthologs of DAF-7 in this parasite [33] and in *S. ratti* and *Parastrongyloides trichosuri* [34]. DAF-7 is the ligand that activates TGF-β-like signaling and thereby promotes continuous (i.e. non-dauer) development in *C. elegans*. Its expression is biased towards *C. elegans* first-stage larvae fated for continuous development rather than dauer third-stage larvae [34], [35]. By contrast, messages encoding DAF-7 orthologs in *S. stercoralis*, *S. ratti* and *P. trichosuri* all show biased expression in the L3i, which has been characterized heretofore as dauer-like [33], [34]. These facts notwithstanding, outright rejection of the ‘dauer hypothesis’ of developmental regulation in the L3i of parasitic nematodes on the basic of transcriptional data alone is likely to be premature [36]. It is particularly noteworthy in this regard that key signal transducing elements such as DAF-16 that directly regulate *C. elegans* dauer development are constitutively transcribed and their functions governed not at the transcriptional level but rather by posttranslational modifications such as phosphorylation [37], [38].

The true value in identifying these and other genetic determinants of *S. stercoralis* parasitism lies in whether the products of these genes can induce protective immunity. Indeed, one of the genes identified in our list, the *S. stercoralis* ortholog of *eat-6 Na+k+ATPase*, has already been identified as a potential vaccine candidate based on animal experiments [39].

### Additional therapeutic targets and immunodiagnostic genes of significance

Contig 1872, a gene with L3i biased expression, encodes an ortholog of *C. elegans* core subunit of the cytochrome bc1 complex, UCR 2.1 (E-value = 1E-014). This subunit has been shown to be a potential target for antiparasitic drugs based on the finding that in *C. elegans*, UCR 2.1 is essential for viability and is less related to mammalian UCR-1 than to mitochondrial processing peptidases from other organisms [40]. *S. stercoralis* transgenesis experiments [41] may prove useful in investigating the question of whether this gene is similarly essential for *S. stercoralis* larval survival.

In our microarray analysis of *S. stercoralis*, we found abundant L3i expression of the *S. stercoralis* ortholog of *hsp-90*, contig_77 (3 fold expression difference). Interestingly, the *hsp-90* inhibitor geldanamycin has been shown to have a macrofilaricidal effect on filarial nematode *Brugia pahangi* [42]. *Hsp-90* has been identified among *S. ratti* parasitism central genes critical for survival and further studies investigating it as a chemotherapeutic target are warranted.

Contig 1151, which was among the 25 most highly biased L3i genes (11-fold expression difference), corresponds to fatty acid and retinol binding protein-1 (FAR-1; E-value = 1E-016). FAR-like proteins are major secreted products of parasitic nematodes that allow the parasite to scavenge essential nutrients from its host [43]. Depletion of host lipids is thought to be necessary for parasite survival and may additionally impair the host immune response [44]. These proteins have additionally demonstrated stage and gender specificity in other nematodes, most notably in the hookworm *Ancylostoma ceylanicum* [45]. The immunodiagnostic potential of FAR-like proteins has been assessed in other nematodes, such as *Onchocerca volvulus*, in a serologic assay based on *Ov-20* (FAR-1) [45], [46], [47]. FAR-1 proteins have been successfully used in a vaccine in animals infected with *A.ceylanicum* [45]. These microarray data identify *S. stercoralis far-1* as an L3i-biased target that may be a potential vaccine candidate or immunodiagnostic antigen.

### Limitations

Approximately one-third of *S. stercoralis* genes are of unknown function. This finding is consistent with a previous EST analysis that revealed a similar percentage (25%) of *S. stercoralis* clusters with no significant BLAST alignments [8]. This finding is also consistent with functional genomics analyses of the *C. elegans* and human genomes where significant numbers of genes of unknown function were identified [48], [49]. Some of these unknown sequences may derive from 3^′^ untranslated mRNA regions, which are common in polydT-primed libraries [50]. The complete genome sequence of *S. stercoralis* is not available to date. Inferred functional annotations of an analogous nematode *C. elegans*, while useful, may not be directly applicable to *S. stercoralis*, as suggested by interspecies differences uncovered in the present comparative microarray analysis. Because a number of *C. elegans* genes did not have *S. stercoralis* orthologs that were also differentially expressed according to our predefined ‘cutoffs,’ it was difficult to formulate gene lists organized into functional categories with at least 5 contigs. This limited our ability to analyze biochemical or metabolic pathways of potential importance. As our knowledge of the *S. stercoralis* genome increases, these microarray analyses will likely gain in usefulness and a more direct approach using annotation based on known *S. stercoralis* gene functions would be even more informative.

### Conclusions

DNA microarrays allow for simultaneous analysis of large numbers of genes from two or more biologic conditions. This powerful method of analysis has revolutionized our understanding of the immunopathogenesis of schistosomiasis [51], for example, and has advanced the development of vaccine discovery and therapeutics in parasitology [52], [53]. Until now, studies of *S. stercoralis* have been limited to the analysis of ESTs rather than the full genome sequence. Development of a novel DNA microarray tool for the study of *S. stercoralis* represents an exciting step forward in our understanding of this parasite.

## Supporting Information

Text S1 This file contains supplemental information regarding microarray probe information (Table S1), primer probe sequences used in real-time PCR analysis (Table S2), all contigs (Table S3), L1 biased contigs (Table S4), L3i biased contigs (Table S5), most highly expressed L1 and L3i contigs (Table S6), L3i biased contigs containing sperm containing glycoprotein domain (Table S7), and results of the GSEA for immunoreactive genes (Table S8) and heat shock proteins (Table S9). For the column marked “Manual Annotation,” the following abbreviations were used: em = energy metabolism; extmat = extracellular matrix; cs = cytoskeleton; imm = genes encoding antigens known to be immunoreactive in sera from patients infected with *S. stercoralis*; met = metabolism; nr = nuclear regulation; pe = protein export machinery; pm = protein modification; prot = proteasome machinery; ps = protein synthesis; st = signal transduction; tf = transcription factor; tm = transcription machinery; tr = transporters and storage proteins; unk = unknown.
(8.05 MB XLSX)
